# Gut Microbiome and Inflammation: A Study of Diabetic Inflammasome-Knockout Mice

**DOI:** 10.1155/2017/6519785

**Published:** 2017-12-31

**Authors:** Roma Pahwa, Miriam Balderas, Ishwarlal Jialal, Xinpu Chen, Ruth Ann Luna, Sridevi Devaraj

**Affiliations:** ^1^Veterans Affairs Medical Center, Mather, CA, USA; ^2^College of Medicine, California Northstate University, Elk Grove, CA, USA; ^3^Texas Children's Microbiome Center, Texas Children's Hospital, Houston, TX, USA; ^4^Department of Pathology and Immunology, Baylor College of Medicine, Houston, TX, USA

## Abstract

**Aims:**

Diabetes is a proinflammatory state, evidenced by increased pattern recognition receptors and the inflammasome (NOD-like receptor family pyrin domain (NLRP)) complex. Recent reports have elucidated the role of the gut microbiome in diabetes, but there is limited data on the gut microbiome in NLRP-KO mice and its effect on diabetes-induced inflammation.

**Methods:**

Gut microbiome composition and biomarkers of inflammation (IL-18, serum amyloid A) were assessed in streptozotocin- (STZ-) induced diabetic mice on a NLRP3-knockout (KO) background versus wild-type diabetic mice.

**Results:**

SAA and IL-18 levels were significantly elevated in diabetic mice (STZ) compared to control (WT) mice, and there was a significant attenuation of inflammation in diabetic NLRP3-KO mice (NLRP3-KO STZ) compared to control mice (*p* < 0.005). Principal coordinate analysis clearly separated controls, STZ, and NLRP3-KO STZ mice. Among the different phyla, there was a significant increase in the Firmicutes : Bacteroidetes ratio in the diabetic group compared to controls. When compared to the WT STZ group, the NLRP3-KO STZ group showed a significant decrease in the Firmicutes : Bacteroidetes ratio. Together, these findings indicate that interaction of the intestinal microbes with the innate immune system is a crucial factor that could modify diabetes and complications.

## 1. Introduction

Diabetes is a worldwide epidemic, and inflammation has been shown to contribute to increased diabetic complications [1]. Diabetes is thus a proinflammatory state characterized by increased C-reactive protein (CRP), proinflammatory cytokines, and monocyte activation [2].

A critical step in the activation of inflammatory responses is the recognition of microbes or endogenous molecules produced in the setting of infection or cellular injury by host pattern recognition receptors [3]. We and others have shown increased pattern recognition receptors, TLR2 and TLR4, in diabetes and have also shown that knockout of TLR2 and/or TLR4 in a mouse model can result in decreased microvascular complications using the streptozotocin- (STZ-) induced diabetes model [4–6]. Another major innate signaling pathway is the inflammasome, a multiprotein platform that activates caspase-1 leading to the proteolytic processing of prointerleukin-1*β* (pro-IL-1*β*) and pro-IL-18 into their mature active forms, IL-1*β* and IL-18 [7]. Among the intracellular Nod-like receptor (NLR) family [8] of inflammasomes, NLRP3 is activated by multiple stimuli including bacterial pore-forming toxins, ATP, microbial RNA, and particulate matter [9, 10]. Several recent reports have elucidated the role of the gut microbiome and its components in the pathogenesis of diabetes and its sequelae [11], and MyD88-KO mice on a NOD background have decreased inflammation and diabetes development [12]. Blocking inflammasome activation has been shown to decrease inflammation and is beneficial in decreasing intestinal inflammation as well as effective in nonalcoholic fatty liver disease (NAFLD) [13].

There is however a paucity of data on the gut microbiome in NLRP3^−/−^ (NLRP3-KO) mice and its effect on diabetes-induced inflammation. In this study, we provide evidence that (i) specific taxa of the gut microbiota present significantly altered prevalences when diabetes is induced by means of STZ in wild-type mice and (ii) these changes are attenuated when diabetes is induced in NLRP3-KO mice, correlating with the decreased inflammation picture that, as expected, characterizes these mice.

## 2. Methods

Wild-type (WT) and NLRP3-KO mice generated on a C57BL/6J genetic background (male, 5 weeks of age, body weight 19–24 g) were purchased from Jackson Laboratory. The protocol was approved by the institution and conforms to the National Institutes of Health Guide for the Care and Use of Laboratory Animals (NIH publication number 8023, revised 1978). The animals were housed in a pathogen-free animal facility with free access to normal chow and water. In these mice, diabetes was induced by intraperitoneal administration of 60 mg/kg STZ freshly dissolved in sodium citrate at pH 4.5 for 5 consecutive days in 7-week-old mice as described before [14]. The 3 groups are referred to as WT or wild type, WT + STZ or diabetic, and NLRP3-KO STZ or diabetic with knockout of NLRP3 (*n* = 6, 9, and 8, resp.). Since there were no observable changes in the wild-type NLRP3-KO mice (*n* = 4), the data are not presented here. Blood glucose levels were measured after 5 days, and diabetes was established with a glucose level of >250 mg/dl on 3 consecutive days. When animals show signs of decompensation defined as progressive weight loss with severe hyperglycemia with glucose levels > 500 mg/dl over 3 days, they were injected with low-dose insulin (1.0 unit subcutaneously) and intraperitoneal Ringer's lactate (0.5 to 1.0 ml). Blood and stool were collected after 24 weeks of diabetes in mice. Before euthanasia, 24-hour urine and fecal collection was performed using metabolic cages. All procedures were approved by the Institutional Animal Care and Use Committees (IACUCs), University of California Davis Medical Center, Sacramento.

### 2.1. Measures of Inflammation

Serum amyloid A, IL-1*β*, and IL-18 in plasma were measured by ELISA (R&D) as per the manufacturer's instructions.

### 2.2. Microbiome Analysis

Approximately 0.02 g of stool was added to a MO BIO PowerBead Tube and processed through the standard MO BIO PowerSoil extraction kit protocol (MO BIO Laboratories, Carlsbad, CA). Quantity and quality of the resulting nucleic acid content were assessed by Nanodrop 1000 and Qubit. Amplification and sequencing of the V1V3 region of the 16S rRNA gene were performed using the NEXTflex 16S V1V3 Amplicon-Seq Kit 2.0 (Bioo Scientific, Austin, TX) with 20 ng of input DNA, and sequences were generated on the Illumina MiSeq platform (Illumina, San Diego, CA) with a minimum of 800 and an average of 7500 sequences generated per sample. Sequence data was processed through the LotuS pipeline as previously described [15]. Sequencing reads were processed through the LotuS pipeline, reads were demultiplexed, and paired ends were stitched. Quality filters using a modified version of the UPARSE algorithm were utilized to reduce error rates [16]. Taxonomic assignment was performed with RDP as the classifier and HitDB and SILVA as the selected databases [17, 18]. Alpha diversity was calculated using QIIME 1.7 for subsequent data processing: Chao1 index and Shannon diversity index. Beta diversity was determined by the unweighted UniFrac distance and Bray-Curtis dissimilarity. OTUs failing to classify bacteria at the kingdom level were removed before further analysis.

Statistical analysis of taxonomic and functional profiles (STAMP) was employed for the visualization and statistical analysis of OTUs [19]. To visualize the distributions of beta diversity values, a PCoA plot was generated using a dissimilarity measure calculated between every pair of community samples with individual dots on the PCoA plot representing distinct microbial communities. Comparisons between groups were made at various taxonomic levels, including the OTU level. Taxonomic assignments for representative sequences of significant OTUs were confirmed by manual database searches and alignments. In addition, microbial differences were evaluated based on other available clinical data, such as glucose and biomarkers of inflammation.

## 3. Results

Baseline characteristics of the mice at 24 weeks are shown in Table 1. There was a small but significant decrease in body weight of diabetic mice. Despite insulin administration, plasma glucose levels continued to be significantly elevated in the STZ mice compared to controls. Although higher plasma glucose levels were observed in NLRP3-KO STZ mice compared to controls, they were not significantly different; the only significant difference was those between WTSTZ and NLRP3-KO STZ mice.

Since NLRP3 inflammasome regulates inflammatory pathways in diabetes, we examined serum amyloid A (SAA), a major prototypic marker of inflammation in rodents, as well as circulating levels of IL-1*β* and IL-18 (Table 2). IL-1*β* levels were below the detection range of the high-sensitivity assay. However, as reported previously in many studies, SAA levels were significantly elevated in diabetic mice and there was a significant attenuation of inflammation in the NLRP3-KO STZ mice to the level of the control mice (*p* < 0.005). Another marker of the activation of the inflammasome complex is IL-18. IL-18 levels were significantly increased in diabetic mice, and knockout of NLRP3 resulted in a significant abrogation of this proinflammatory cytokine in diabetic mice.

**Figure 1:**
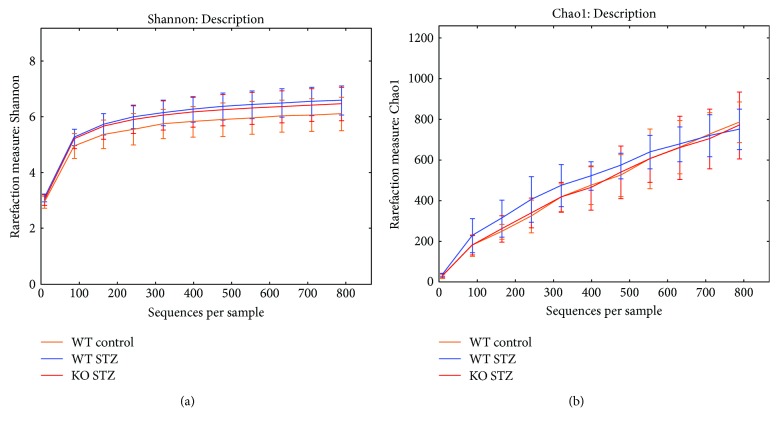
(a) Rarefaction curves demonstrating the Shannon index (evenness and richness metric) of the OTUs identified for the three groups: WT control, WT STZ, and NLRP3-KO STZ. Rarefaction of the OTU table was set to 794. Curves suggest no significant differences in alpha diversity. (b) Rarefaction curve using the Chao1 index for the three groups: WT control, WT STZ, and NLRP3-KO STZ, calculated using QIIME 1.7. Rarefaction of the OTU table was set to 794. Curves suggest no differences in alpha diversity.

**Figure 2:**
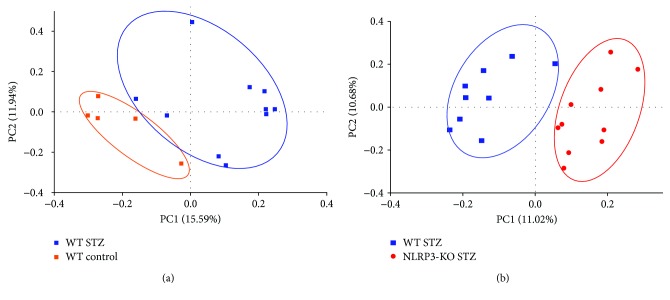
(a) PCoA plot of the WT group by unweighted UniFrac distances. Analyses were determined using QIIME 1.7. The axes represent the first highest discriminating axes using the Bray-Curtis distance measure. (b) PCoA plot of the treatment groups by unweighted UniFrac distances. Analyses were determined using QIIME 1.7. The axes represent the first highest discriminating axes using the Bray-Curtis distance measure.

Lastly, we examined the effect of the NLRP3 inflammasome and inflammation on gut microbiota composition. The examination of alpha diversity of the gut microbiota as depicted by the Shannon or Chao index (Figures 1(a) and 1(b)) showed no significant differences between wild-type control, STZ, and NLRP3-KO STZ groups. Since there were no observable changes in the wild-type NLRP3-KO mice (*n* = 4), the data are not presented here. Principal coordinate analysis of 16S sequences from samples using UniFrac shows a distinct separation of sample groups of controls (orange) and STZ (blue) (Figure 2(a)) as well as a distinct separation between STZ (blue) and NLRP3^−/−^ STZ (red) (Figure 2(b)).

**Figure 3:**
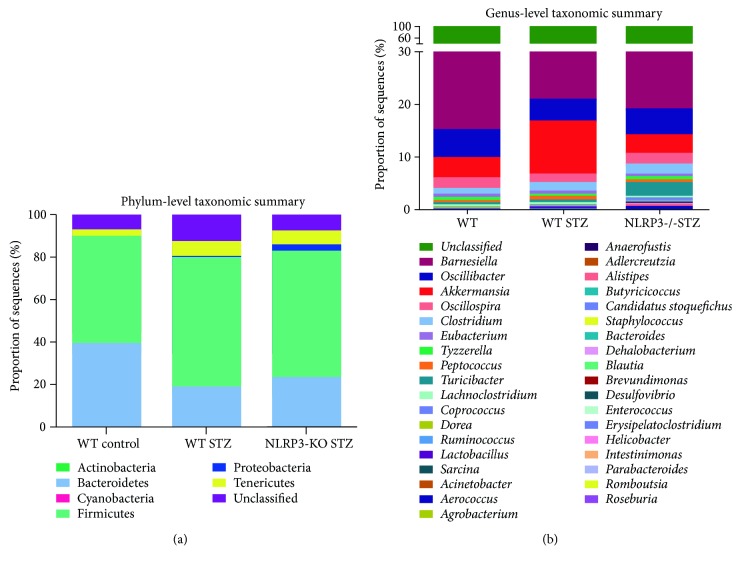
(a) The taxonomic distribution of the WT control, WT STZ, and NLRP3-KO STZ groups is represented as the percent abundance of the identified phyla for each group. Analyses were determined using QIIME 1.7, and raw data was entered into GraphPad Prism to generate a stacked bar plot. (b) The taxonomic distribution of the WT control, WT STZ, and NLRP3-KO STZ groups is represented as the percent abundance of the identified genera for each group. Analyses were determined using QIIME 1.7, and raw data was entered into GraphPad Prism to generate a stacked bar plot.

Next, taxonomy was assigned on the three groups and represented as percent abundance at the level of bacterial phyla (Figure 3(a)) and genera (Figure 3(b)). Among the different phyla, there were a significant increase in Firmicutes and a decrease in Bacteroidetes in the STZ–diabetic group compared to WT controls. Also, there was a significant increase in the Firmicutes : Bacteroidetes ratio in WT STZ mice which was restored in the NLRP3-KO STZ mice (WT: 1.25 ± 0.29; STZ: 3.0 ± 1.1; NLRP3-KO STZ: 2.2 ± 0.91; *p* < 0.01 for the trend). When compared to WT STZ, the NLRP3-KO STZ group showed a significant increase in Bacteroidetes and a nonsignificant decrease in Firmicutes. The remainder of the microbiota was composed of divisions commonly encountered at lower abundance in the mouse and human gut: Verrucomicrobia, Proteobacteria, Cyanobacteria, Actinobacteria, and the candidate phylum TM7. At the genus level, there were increased *Akkermansia* and decreased *Barnesiella* and *Oscillibacter* in STZ which was reversed in diabetic NLRP3-KO.

## 4. Discussion

The results presented in this report provide evidence that modulation of the intestinal microbiota through the regulation of the NLRP3 inflammasome is important in regulating inflammation in diabetic animals and has an effect on glucose homeostasis.

Recent reports suggest a complex role of inflammasome function in multiple manifestations of the metabolic syndrome. Activation of IL-1*β*, mainly through cleavage by the NLRP3 inflammasome, promotes insulin resistance [20]. While the gut microbiota is important in diabetes, there is a paucity of data on the relationship of inflammation and microbiota in diabetes and its complications. In this report, in addition to decreased inflammation in the NLRP3-KO mice in the diabetic milieu compared to STZ mice, we show a significant increase in Firmicutes and a decrease in Bacteroidetes in the STZ–diabetic group compared to WT controls and an associated significant increase in the Firmicutes : Bacteroidetes ratio in STZ which was restored in the NLRP3-KO STZ mice.

Previously, Xie et al. [21] showed that compared to controls, STZ mice on a high-fat diet had fewer Bacteroidetes and Proteobacteria but higher levels of Firmicutes, Tenericutes, and Actinobacteria. In concordance with those studies, we also report a significant increase in Firmicutes and a decrease in Bacteroidetes in the STZ–diabetic group compared to WT controls. Also, there was a significant increase in the Firmicutes : Bacteroidetes ratio in STZ which was restored in the NLRP3-KO. A change in the Firmicutes : Bacteroidetes ratio by itself may be important since it can influence the processing of dietary polysaccharides [22, 23]. In this context, our findings are important since it has been previously shown that deficiency of the TLR adaptor protein, MyD88, changes the composition of the distal gut microbiota as well as the exposure of the microbiota to SPF NOD. MyD88-negative donors attenuate type 1 diabetes in germ-free NOD recipients [12]. Furthermore, NLRP6^−/−^ mice have been demonstrated to have reduced IL-18 and increased representation of Prevotellaceae which belongs to the Bacteroidetes phyla [24], and this lends support to our findings in NLRP3^−/−^ mice on a diabetic background. Genus-level-specific findings from our study need to be corroborated with results from larger studies.

## 5. Conclusion

Together, these findings indicate that interaction of the intestinal microbes with the innate immune system and resulting inflammation is a crucial factor that could modify diabetes and its complications. Understanding precise signals that activate each of the inflammasomes and thereby regulate the gut microbiome composition would be important for developing therapeutic strategies in diabetes and its complications.

## Figures and Tables

**Table 1:** Baseline characteristics.

	WT (*n* = 5)	WT-STZ (*n* = 10)	NLRP3-KO STZ (*n* = 9)
Body weight (g)	35.1 ± 3.1	28.9 ± 2.5^∗^	30.5 ± 3.1
Plasma glucose (mg/dl)	181.3 ± 48.5	916.3 ± 482.6^∗^	393.7 ± 310.3^∗**#**^

Data are presented as mean ± SD. ^∗^*p* < 0.05 versus WT. ^#^*p* < 0.05 versus WT-STZ.

**Table 2:** Biomarkers of inflammation.

	WT (*n* = 5)	WT-STZ (*n* = 10)	NLRP-KO STZ (*n* = 9)
SAA (*u*g/ml)	56 (45, 66)	110 (91, 132)^∗^	50 (19, 63)^#^
IL-18 (pg/ml)	18 (15, 22)	71 (57, 88)^∗^	54 (12, 64)^#^

Data are presented as median (25th percentile, 75th percentile). ^∗^*p* < 0.05 versus WT. ^#^*p* < 0.005 versus WT-STZ.
